# Engineering of Fc Multimers as a Protein Therapy for Autoimmune Disease

**DOI:** 10.3389/fimmu.2020.00496

**Published:** 2020-03-25

**Authors:** Elizabeth A. Fitzpatrick, Jin Wang, S. E. Strome

**Affiliations:** ^1^Department of Microbiology, Immunology and Biochemistry, College of Medicine, University of Tennessee Health Science Center (UTHSC), Memphis, TN, United States; ^2^College of Graduate Health Sciences, University of Tennessee Health Science Center, Memphis, TN, United States

**Keywords:** IVIG—intravenous immunoglobulin, Fc multimer, autoimmune, FcgR, complement

## Abstract

The success of Intravenous Immunoglobulin in treating autoimmune and inflammatory processes such as immune thrombocytopenia purpura and Kawasaki disease has led to renewed interest in developing recombinant molecules capable of recapitulating these therapeutic effects. The anti-inflammatory properties of IVIG are, in part, due to the Fc region of the IgG molecule, which interacts with activating or inhibitory Fcγ receptors (FcγRs), the neonatal Fc Receptor, non-canonical FcRs expressed by immune cells and complement proteins. In most cases, Fc interactions with these cognate receptors are dependent upon avidity—avidity which naturally occurs when polyclonal antibodies recognize unique antigens on a given target. The functional consequences of these avid interactions include antibody dependent cell-mediated cytotoxicity, antibody dependent cell phagocytosis, degranulation, direct killing, and/or complement activation—all of which are associated with long-term immunomodulatory effects. Many of these immunologic effects can be recapitulated using recombinant or non-recombinant approaches to induce Fc multimerization, affording the potential to develop a new class of therapeutics. In this review, we discuss the history of tolerance induction by immune complexes that has led to the therapeutic development of artificial Fc bearing immune aggregates and recombinant Fc multimers. The contribution of structure, aggregation and N-glycosylation to human IgG: FcγR interactions and the functional effect(s) of these interactions are reviewed. Understanding the mechanisms by which Fc multimers induce tolerance and attempts to engineer Fc multimers to target specific FcγRs and/or specific effector functions in autoimmune disorders is explored in detail.

## Introduction

Immunoglobulin (IVIG) is approved as a therapeutic for chronic autoimmune and inflammatory processes such as immune thrombocytopenia purpura (ITP) and Kawasaki disease, among others (1). However, IVIG is expensive to produce, has blood borne pathogen risks, toxic side effects, and because it is pooled from plasma from thousands of human donors, there is both a lack of consistency among preparations (2–5) and intermittent supply shortages. There is a critical need to develop recombinant therapeutics that reproduce the anti-inflammatory effects of IVIG. One of the major mechanisms by which IVIG exerts anti-inflammatory properties is through the Fc domain of the IgG molecule (6). The Fc fragment has important biological effector functions that are controlled by IgG isotype, aggregation, interactions with FcγRs and complement components. Identifying the mechanisms by which these factors contribute to the protective effect of IVIG is critical to the development of novel IVIG replacement therapies.

## Structure of IgG And Fc Fragment

**Figure 1:**
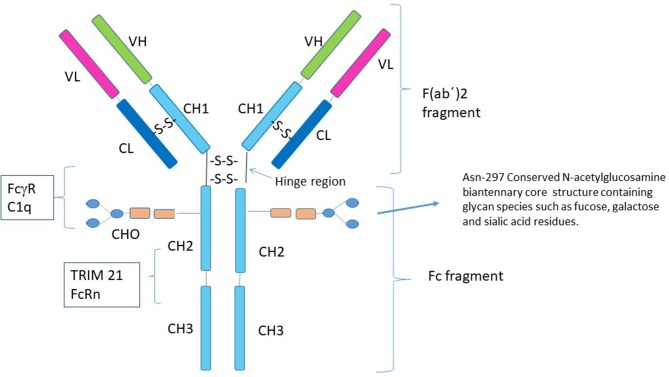
Structure of IgG1. Human IgG consists of four subclasses (IgG1–IgG4) that differ in the number of disulfide bonds in the hinge region, amino acid residues throughout the constant chain as well as their glycosylation patterns. Although the conserved N-linked glycosylation site at Asn-297 is necessary for stabilization of the Fc fragment and its removal abolishes Fc effector functions of IgG (7–9), it is not absolutely required for the anti-inflammatory activity of Fc multimers. Residues near the lower hinge region, C_H_2 and C_H_3 are involved in binding to FcγRs and C1q. The FcRn and TRIM21 bind to residues within both the C_H_2 and C_H_3 region. The majority of studies with recombinant Fc proteins for use as replacement IVIG have focused on introducing sequences that allow for aggregation of the recombinant Fc molecules, for example addition of a IgG2 hinge region as a multimerization domain to the IgG1 Fc (10, 11).

The immunoglobulin molecule (IgG) consists of two identical light chains and two identical heavy chains that can be divided into two proteolytic fragments; the antigen-binding fragment (Fab) and the Fc fragment consisting of the C_H_2 and C_H_3 regions of the heavy chain (Figure 1). The Fc fragment mediates effector functions of IgG such as antibody-dependent cellular cytotoxicity (ADCC) or complement-dependent cytotoxicity (CDC), through the binding of soluble and cell-surface proteins to distinct residues within the C_H_2 and C_H_3 domain. The classical FcγRs bind to residues near the hinge region in C_H_2 and have a partial overlap with the site of C1q binding (12–17). Additional Fc binding proteins such as the neonatal Fc receptor (FcRn) and Tripartite motif-containing 21 (TRIM21) bind to residues within both the C_H_2 and C_H_3 region (18, 19). There is considerable heterogeneity in Fc glycosylation and the amino acid sequences of hinge regions between different IgG isotypes, all of which affect the binding affinity of IgGs to FcγR's and consequent effector function.

### Glycosylation

Within the C_H_2 portion of the Fc fragment is a conserved N-linked glycosylation site at Asn-297, which consists of a mannose and N-acetylglucosamine (GlcNAc) core structure and varying glycan species including fucose and galactose that may have added sialic acid residues (20). The N-linked glycan, N297, is necessary for stabilization of the Fc fragment and binding to FcγRs and its removal abolishes Fc effector functions (7). Changes in the patterns of N-linked glycosylation are recognized to affect the biologic properties of antibodies (21–24). Particularly germane to this review, are studies suggesting that α 2,6 sialylated Fc fragments are responsible for all of the anti-inflammatory properties of IVIG through engagement of the C-type lectin DC-SIGN (25). In support of this theory, several reports now suggest that the degree of circulating sialylated antibodies correlates with the activity of specific autoimmune and inflammatory conditions in humans (26–28). However, a large cohort of other studies clearly refute the import of α 2,6 sialylated Fc fragments in the function of IVIG mediated tolerance, showing that these fragments do not bind DC-SIGN and that removal of α 2,6 sialylated Fcs does not impact the anti-inflammatory properties of IVIG (29–32). As such, the role of α 2,6 sialylated Fcs in the tolerogenic properties of IVIG remains the subject of ongoing scientific debate.

## FcγR's

**Figure 2:**
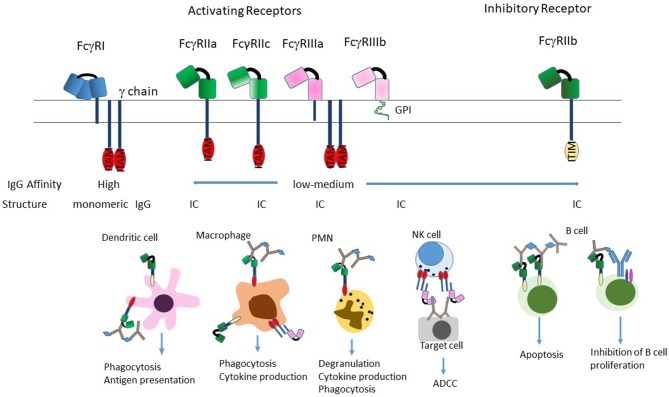
Structure of FcγRs. The FcγR's differ in their affinity for IgG; FcγRI is a high-affinity receptor and is the only one that can effectively bind monomeric IgG; the two low-affinity receptors FcγRII and FcγRIII preferentially bind IgG in the form of immune complexes. FcγRI and FcγRIIIa exist as transmembrane proteins each non-covalently linked to a common FcRγ subunit. The γ subunit exists as a homodimer containing an immunoreceptor tyrosine-based activation motif (ITAM) within its intracellular domain. FcγRII exists on the cell surface as a single chain with the ligand-binding region in the extracellular domain and either an ITAM (FcγRIIa), or an immunoreceptor tyrosine-based inhibition motif (ITIM; FcγRIIb) in the intracytoplasmic domain necessary for signal transduction reviewed in (33). FcγRIIIb is the only receptor anchored to the membrane via a glycosylphosphatidylinisotol (GPI) link (34). Stimulation of the FcγRs by ICs induces a variety of effector functions that varies by cell type. Most cells express a combination of activating and inhibitory receptors, which allows fine-tuning of the response to ICs. The exception to this is NK cells, which only express activating receptors, and B cells that only express the inhibitory receptor.

The classical FcγRs are cell membrane associated proteins expressed on a variety of immune cells such as macrophages, dendritic cells (DCs), natural killer (NK) cells, neutrophils, and B cells. In humans, there are three types of FcγRs: hFcγRI /CD64, hFcγRII/CD32, and hFcγRIII/CD16, that are grouped based on structural homology (Figure 2). The FcγRII and FcγRIII subfamilies are further subdivided into FcγRIIa (CD32a), FcγRIIb (CD32b) and FcγRIIc (CD32c) and FcγRIIIa (CD16a) and FcγRIIIb (CD16b). From a conceptual perspective, the functional differences in these individual FcγRs are based on their different affinities for the naturally occurring Fc fragment, their inducibility, their patterns of cellular expression, their ability to mediate internalization of immune complexes and the pathways through which they signal [reviewed in (33, 35, 36)].

## Other IgG Fc Binding Ligands

Complementing the classical FcγRs, are a relatively diverse set of non-canonical FcγRs which contribute to the functions of antibody homodimers and Fc bearing ICs. For instance, the neonatal FcR (FcRn) is an MHC Class I–like molecule that is associated with β2-microglobulin and is responsible for IgG half-life as well as other functions such as transferring IgG from the mother across the placenta to the fetus [reviewed in (37–43)]. Similarly, intracellular receptor Tripartite motif-containing 21 (TRIM21) appears to play a role in neutralization of antibody decorated pathogens (19, 44–48)]. Finally, FcRL5 is a cell surface protein expressed on B cells and able to bind all IgG subclasses (49, 50). It has two ITIM and two ITAM motifs within its cytoplasmic domain suggesting that it can induce inhibitory or activating signals (51).

In addition to these cell-based receptors, soluble proteins such as complement can also engage multimerized/aggregated Fc. For instance, C1q is a hexamer composed of two trimers containing an A, B, and C chain each with collagen-like stalk portions and globular heads resulting in the characteristic “bundle of six tulips” (52). Binding of C1q by ICs results in the engagement of C1r and C1s and subsequently activation of the classical complement cascade. (53–56). Importantly, and as discussed in later sections, unlike the scenario in which C1q binds an antibody decorated cell and induces C5b-9 pore formation, IC mediated activation of the classical cascade may occur away from the cell surface, resulting in a poorly defined series of immunomodulatory effects.

## Conceptual Basis for the Development of Fc Multimers to Induce Tolerance

The conceptual basis for developing Fc multimers as a tolerogenic therapy is based on the anti-inflammatory role of the Fc fragment in IVIG. However, there is historical evidence that ICs possess anti-inflammatory properties in addition to their well-described pro-inflammatory effects. Careful observational studies by Flexner in 1906, introduced the concept of tumor enhancement, where heated tumor emulsions administered 10 days prior to tumor implantation, augmented the growth of subsequent tumor implants in rats (57). Although any role of IgG in these earlier studies is unclear, the phenomenon of tumor enhancement has been observed following passive transfer of anti-serum prior to tumor inoculation (58). Studies by Kaliss et al. (59–61) demonstrated that transfer of serum from animals that had rejected a primary tumor to naïve animals potentiated subsequent tumor growth. The enhancing activity was found to be associated with the gamma-globulin fraction (62). Many mechanisms have been put forward to explain tumor enhancement by IgG including masking of tumor antigens by antibody (61, 63) or a shift in the Th1 to Th2 cytokine response favoring tumor growth (64), however FcγR dependent mechanisms such as inhibition of ADCC may also explain this phenomenon (63).

Clinical evidence supporting the potential therapeutic role of ICs in preventing disease is derived from the observation that treatment of ITP patients with anti-D antibodies results in an increase in platelet counts (65–67). The fact that treatment efficacy is dependent on patients being Rh^+^, suggests that these antibodies function by presenting aggregated Fc on the surface of Rh^+^ cells (68, 69). The observation that it requires at least 48 h for patients to respond—longer than the time necessary for saturation of the FcR—highlights the immunomodulatory nature of these effects (68).

The conceptual underpinnings for the idea that Fc multimers have anti-inflammatory properties are also founded on examples of naturally occurring multimeric Fc like proteins with tolerogenic properties. For instance, the short pentraxins are evolutionarily conserved precursors of existing antibodies, whose pentameric structure allows them to engage the low affinity FcγRs and complement (70). While initially recognized to bind and regulate the immune response to specific pathogens and apoptotic cells, recent data suggest that these proteins also have profound anti-inflammatory activity [reviewed in (71)]. For example, serum amyloid P (SAP) inhibits many of the pro-inflammatory components of neutrophil function and also prevents the conversion of monocytes into fibrocytes, potentially mitigating fibrosis (72). Collectively, these studies provide both the historical context and biologic basis for employing recombinant multimerized Fc compounds to mimic the tolerogenic properties of the Fc portion of aggregates in IVIG. In addition, they force a re-evaluation of the concept that ICs only induce inflammation—suggesting that in some cases they may be induced in response to pro-inflammatory events as a means to restore immune homeostasis.

## Development of Recombinant Fc Multimers as Therapeutics

Based on the supposition that artificial Fc bearing immune complexes might induce tolerance, our laboratory in collaboration with Gliknik®, followed by several other groups, sought to develop fully recombinant IgG multimers for both clinical translation and mechanistic experiments. Specifically, we developed linked multimerization domain (MD) sequences from the hinge region of human IgG2 or the isoleucine zipper (ILZ) to the carboxy or amino termini of the murine IgG2a. The resultant stradomers™ contained both homodimers and highly ordered multimers of the Fc homodimers. One of these stradomers™, bearing the IgG2 hinge (M045), effectively binds to FcγRI, FcγRIIb and FcγRIII with significantly lower Kd values than control IgG2a Fc, inhibits the development of collagen-induced arthritis (CIA) and protects against platelet destruction. The fact that most of the therapeutic activity of this drug resides in the multimeric fraction, highlights the relative importance of avidity, rather than affinity, for its biologic activity (10). Collectively, these data provided the first evidence that *recombinant* immune complexes could induce tolerance.

Subsequent studies confirmed and extended these findings, demonstrating that stradomers™ can effectively inhibit development of experimental autoimmune neuritis model (73) and experimental autoimmune myasthenia gravis (EAMG) (74). Importantly, the studies in EAMG provided significant mechanistic insights, showing that daily administration of stradomers™ reduces Acetylcholine Receptor (AchR) antibody levels, decreases antigen specific T cell proliferation, down-modulates both B cell and DC maturation markers, up-regulates inhibitory FcγRIIb expression, and is associated with an increase in both Tregs and immunosuppressive cytokines such as IL-10 and IL-4 (74). We are attempting to distinguish the relative importance of the FcRs and complement on these biologics by employing complement preferential stradomers^TM^ *in vitro* and *in vivo* (11, 75).

In order to translate our preclinical findings, we developed a human analog of these drugs, GL-2045, by joining the human IgG2 hinge region to the C-terminus of the *human* IgG1 Fc fragment (76). GL-2045 avidly binds human FcγRI, FcγRIIa, FcγRIIb and FcγRIIIa as well as to rat, mouse and cynomolgus monkey FcγRs, protects mice from platelet loss in a rodent ITP model and inhibits CIA. Of perhaps greater import, GL-2045 infusion into healthy cynomolgus monkeys is well-tolerated and induces transient and highly ordered increases in IL-1RA and IL-10 as well as a temporary suppression of IL-8, without significant induction of proinflammatory cytokines (76).

Following our initial studies, several other groups reported that recombinant Fc multimers can ameliorate autoimmune disease, suggesting that some of the properties of these multimers might be generalizable. For instance, Mekhaiel et al. (77) generated a hexameric Fc by joining the Fc portion of human IgG1 to an 18 amino acid sequence from the C-termini of the IgM μ-tailpiece with a leucine 309 to a cysteine mutation (78, 79). This compound exhibits greater affinity for the FcγRs than IVIG and upon internalization, is associated with preferential degradation of the *activating* FcγRs and protects mice from platelet loss for up to 3 days after dosing (80). Studies with analogous compounds demonstrate clinical efficacy in both CIA and in the K/BxN model of chronic arthritis (81). These data lend credence to the idea that structurally distinct ICs can have anti-inflammatory properties.

In order to better understand the relationship between IgG1 Fc valency/ structure on FcγR engagement/function, Ortiz et al. (82) evaluated the function of Fc multimers with increasing valency and observed that structures containing 2 and 3 Fc domains avidly bind FcγRs, but unlike molecules containing 5 Fc domains, do not induce Syk phosphorylation or a calcium flux in macrophages. In addition, the larger structures are internalized along with FcγRII, whereas the smaller structures remain on the cell surface co-localized with FcγRII. Subsequent studies showed that the trivalent Fc (Fc3Y) competitively inhibits several IC mediated FcγR functions and protects mice from ITP (82). Importantly, given the valency of Fc3Y, the extent to which it can immunomodulate the complement cascade is uncertain. Collectively, these data support the idea that Fc bearing immune complexes may serve as a protective mechanism against inflammation and, as a corollary, that recombinant Fc multimers might have therapeutic value for the treatment of autoimmunity.

## Anti-Inflammatory Mechanisms of Recombinant Fc Multimers

The development of Fc multimers as a replacement for IVIG is a significant therapeutic advance. Importantly, like IVIG, these molecules likely function by numerous overlapping mechanisms, influenced by the number of IgG Fc domains presented to ligands, the IgG isotype, and the conformational flexibility of the aggregate or Fc multimers (76, 77, 82). Moreover, the relative activity is likely dependent upon the specific underlying disease, the state of maturation of immune cells in that disease and other prior treatments.

From a conceptual perspective, it is our hypothesis that recombinant ICs require and/or benefit from immune activation as a necessary precursor for the induction of tolerance. Specifically, given that multiple redundant immunologic pathways are in place to restore immune homeostasis, it is highly possible, if not likely, that the initial inflammatory response induced by recombinant Fc multimers induces a compensatory response that restores immune homeostasis. In this regard, it is noteworthy that IVIG can be associated with initial fever, chills, headache, and transient release of pro-inflammatory cytokines (83, 84) and that hypotension with IVIG administration is related to the rate of infusion of aggregates (85). It is possible that patients receiving IVIG who experience these initial pro-inflammatory effects are the most likely to be subsequently induced into tolerance by the Fc aggregates in IVIG. While this hypothesis remains to be validated, it is conceptually helpful in understanding the potential links between the diverse immunologic alterations mediated by these drug candidates.

### Biologic Decoys and FcγR Blockade

Immune complexes are associated with the development of autoimmune disease and resultant tissue damage. Because naturally occurring ICs are commonly generated around a pathogen or other type of foreign protein, it is difficult to separate the biologic effects of IC: FcR and/or complement engagement from those mediated by the target protein. Using ICs generated around single stranded DNA or RNA (ssDNA/RNA) as an example, the Fc:FcR interactions allow ssDNA/RNA access into the cell where they can drive TLR-mediated inflammation. Lacking ssDNA/RNA, recombinant Fc multimers competitively inhibit natural ICs from FcR engagement (76, 80, 86), preventing subsequent engagement of intracellular TLRs and potentially inducing other active inhibitory functions as a result of FcR stimulation in a non-inflammatory milieu.

Additionally, FcγR's efficiently internalize ICs, which allows for processing and presentation of antigenic peptides to T cells, further amplifying an immune response. The lack of “core” antigen in the Fc multimer preparations is likely key to the ability of the multimers to block FcγRs without inducing further inflammation.

### FcRn Blocking

The FcRn has a documented role in antibody-mediated autoimmunity. Indeed, FcRn deficient mice are protected from serum transfer-induced arthritis (87). Doses of IVIG that resulted in saturation of the FcRn inhibited development of arthritis in mice lacking the inhibitory FcγRIIb (87). The FcRn plays a critical role in maintaining the plasma concentration of IgG such that high concentrations result in increased IgG clearance (88–91). Infusion of high dose IVIG results in a reduction of circulating autoantibody, which may be due in part to saturation of the FcRn and enhanced IgG catabolism (92–95), although it is likely other mechanisms such as the FcγRIIb engagement also play a role (96). It is likely that Fc multimer binding to FcRn may competitively inhibit engagement of circulating antibodies—pathogenic and otherwise—resulting in lysosomal degradation and non-specific decreases in antibody half-life. While many of the recombinant Fc multimers and hexamers bind FcRn *in vitro*, further studies are required to accurately characterize the role of the FcRn in mediating their anti-inflammatory activity.

### Stimulation of the FcγRIIb Inhibitory Receptor

The FcγRIIb inhibitory receptor is the only FcγR expressed by B cells and crosslinking of the B cell receptor (BcR) and FcγRIIb suppresses B cell proliferation and activation (97, 98). In contrast, ligation of FcγRIIb alone may induce B cell apoptosis (99, 100). In addition to directly stimulating the inhibitory receptor on B cells, ICs and IVIG also induce its expression on both B cells and myeloid cells (25, 101, 102). Myeloid cells express both inhibitory and activating receptors and the ratio of inhibitory to activating receptor stimulation will dictate the outcome of signaling. Therefore, IVIG or Fc multimer induced FcγRIIb upregulation may favor inhibitory signaling pathways over the activating pathways.

### Stimulation of Activating FcγRs

The suppressive effects of IVIG (and subsequently Fc multimers) may also be mediated by stimulating the activating receptors. For example, Park-Min et al. (103) demonstrated that IVIG suppresses IFNγ mediated phosphorylation of Stat1 and IFNγ-dependent gene expression in macrophages *in vitro*. *In vivo*, IVIG treatment of mice infected with *Listeria monocytogenes* results in increased bacterial burden and decreases expression of IFNγ-dependent genes IP-10 and MIG. This effect is FcγRIII dependent, as ICs do not inhibit IFNγ signaling in B cells (which only express FcγRIIb), inhibit signaling in NK cells and DCs that express FcγRIII, but fail to inhibit signaling in macrophages deficient in FcγRIII (103).

Stimulation of activating receptors may also modulate the production of anti- and pro-inflammatory cytokines. For instance, ICs induce the production of anti-inflammatory cytokines such as IL-10 (104) and suppress IL-12 production (105, 106). Ligation of the FcγR on macrophages during stimulation with LPS results in a decrease in IL-12 production and an increase in IL-10 compared to LPS stimulated controls, whereas other cytokines remain unchanged (106). The inhibition of IL-12 production is not due to the expression of IL-10 as IL-10^−/−^ macrophages also exhibit decreased IL-12 production following FcγR ligation and LPS stimulation. Taken together, these studies suggest that stimulation of the activating receptors by IVIG or Fc multimers, in the absence of pro-inflammatory signals, may result in induction of tolerance.

### Expansion of Regulatory T Cells

Patients undergoing IVIG treatment exhibit an expansion in Tregs which may contribute to its therapeutic effects (107–109). Fc multimers are recognized to cause similar increases in Tregs, with both GL-2045 and IVIG inducing Treg expansion in the EAMG model (74). An intriguing mechanism by which IVIG (and Fc multimers) may induce Tregs is the presence of highly conserved Tregitopes in the C_H_2 domain of the Fc fragment of human IgG (110). Upon uptake by antigen presenting cells, Tregitopes bind with high affinity to HLA molecules and cause the activation and expansion of Treg cells (111). Additional potential mechanisms for IC mediated Treg expansion include the induction of tolerogenic DCs (112–114). For instance, Trinith et al. (115) demonstrated that *in vitro*, IVIG pre-treatment of DCs results in the differentiation and expansion of Tregs that is dependent on COX-2 induced PGE_2_ production (115). Collectively, these data suggest that IVIG and ICs have the potential to induce Tregs through multiple independent pathways.

### Complement Engagement

**Figure 3:**
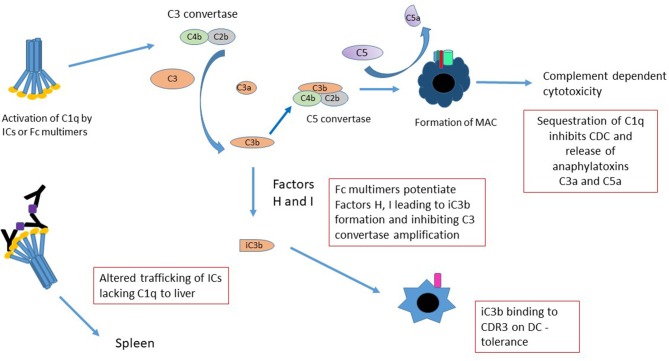
Mechanisms of Fc multimer inhibition of complement function. There are several mechanisms by which Fc multimers may inhibit complement functions. Sequestration of C1q by multimers prevents pathogenic IC-induced CDC, release of anaphylatoxins C3a and C5a and redirects trafficking of C1q-bearing ICs from the spleen to the liver. Activation of complement by Fc multimers leads to limited production of C3 convertase and in the presence of Factor H and I degrades C3b to iC3b (75) inhibiting the C3 convertase amplification loop and potentially increasing iC3b binding to CD3 on DCs inducing tolerance.

While the complement cascade has historically been viewed as pro-inflammatory—with the generation of “anaphylatoxins” such as C3a, more recent studies suggest that it be considered as immunomodulatory (116–118), with the ability to induce long-term tolerance following activation. For example, following cleavage, unbound C3b is very rapidly converted into iC3b; iC3b is capable of binding CR3 on dendritic cells and inducing long-term tolerance (119–121). Sohn et al. (117) demonstrated that iC3b binds to CR3 and modulates IL-10 and TGF-β2, preventing delayed type hypersensitivity (DTH) in a rat ocular DTH model. The fact that stradomers^TM^ that preferentially bind complement (e.g. G211 and likely others) effectively induce iC3b, provides a strong associative link between the generation of this and potentially other anti-inflammatory complement associated molecules and Fc multimer mediated tolerance (Figure 3) (11).

Similarly, indirect evidence suggests that Fc multimers interact with specific regulatory proteins to define the extent of complement activation. For instance, in human serum, G211 and others, mediate cleavage of C4 and to a lesser degree C3, but not significant amounts of C5, as evidenced by the generation of C4a, C3a, and the absence of C5a (11, 75). These effects are abrogated in Factor H deficient sera, suggesting that GL-211 potentiates factor H and/or co-factor I, limiting the generation of C5b-9 and associated cell death.

The classical arm of the complement cascade is generally activated on the cell surface as a result of antibody opsonization. Such activation mediates direct cell lysis through C5b-9 pore formation. In addition, these complement split products; decorating the cell surface, function as biologic bridges to engage complement receptors on other cells types. In contrast, hexameric C1q binding to IVIG or to recombinant Fc multimers, induces formation of C4 protease and cleavage of C4 away from the cell surface. C3 convertase is formed in solution, away from the cell surface, with cleavage of C3 to C3a and C3b which immediately degrades in solution to iC3b. These drugs thereby sequester complement substrates and competitively inhibit their ability to perform other biologic functions (75). Importantly, while this activation may result in soluble C5b-9 formation *in vivo*, soluble C5b-9 is not pore-forming, is not cell-bound, and is not associated with Complement Dependent Cytotoxicity (CDC). Indeed, the exact biologic functions of non-membrane bound C5b-9 remain unclear and may differ from the biology of cell-bound complement activation.

Finally, the act of binding of C1q and other complement products to aggregated immunoglobulins or to multimerized Fcs, alters both the ability of these compounds to engage FcRs and the C1q-mediated trafficking of ICs to liver and spleen. C1q-opsonized ICs bind CR1—located on RBCs in humans and on platelets in mice—where they are then preferentially transported to the spleen (122). When ICs are bound to C3b, they have a limited ability to engage FcγRs, likely because of the partially overlapping binding sites between these moieties (123, 124). In the absence of C1q and/or in instances where the multimers have valency insufficient for C1q binding, they bypass the spleen and are preferentially transported to the liver (125). How/whether the changes in binding and trafficking mediated by complement engagement of Fc multimers alters their biology is an important subject for future study.

## Pro-vs. Anti-Inflammatory Effects of Immune Complexes: A Resolvable Paradox?

Immune complexes can have pro-inflammatory activity—activity which may be conveniently classified in substrate dependent and substrate independent effects. Substrate dependent inflammation is induced by the partnership between FcR/complement engagement and the nidus of the IC. For example, in the case of ICs based on RNA viral substrates, FcR engagement may facilitate trafficking to intracellular TLR7, with resultant inflammation (126, 127). In contrast, substrate independent effects are induced independent of antigen, resulting from binding to the activating FcRs/complement with potential cytokine production, degranulation, cytotoxicity and/or complement activation [reviewed in (128)]. Because recombinant Fc multimers can engage FcRs and select complement fragments in the absence of an “antigenic core” they, by definition, competitively inhibit substrate dependent, Fc mediated inflammation, while promoting substrate independent inflammation in a valency dependent fashion. The degree to which such substrate independent inflammation is required for the generation of tolerance is the subject of ongoing investigation.

### Alternative Therapeutic Strategies for Targeting FcγRs

To our knowledge, there are no clinically relevant chemical based strategies to engineer Fc multimers, However, as an alternative to recombinant Fc multimers, investigators are employing antibodies to target specific FcRs with therapeutic intent. For instance, several groups have generated humanized monoclonal antibodies directed against the inhibitory FcγRIIb (129, 130) as well as the activating receptors FcγRI (131) and FcγRIIIa (132, 133) in an attempt to modulate disease activity. In addition, ongoing clinical trials are evaluating the potential of antibodies targeting the FcRn to reduce systemic antibody half-life and the absolute quantity of circulating antibodies (134, 135). These studies will likely define the precise roles of specific Fc receptors in different disease states.

## Antibodies as Controls

While not the primary focus of this manuscript, the creation of recombinant Fc multimers highlights biologic principles that must be considered when using non-specific IgG as controls for antibody-based studies. Specifically, binding of multiple antibodies to their cognate targets on the surface of a cell, pathogen or other molecule enables their associated Fc fragments to engage low and intermediate Fc receptors and complement. Importantly, this engagement occurs at the site dictated by the location of the epitope recognized by the Fab. In contrast, because isotype specific antibody controls lack the ability to engage epitopes they will have a different distribution within the patient/animal as the experimental antibody and not engage similar Fc receptors or cell types as the experimental antibody. In addition, the IgG isotype controls will not form IC and therefore will likely engage higher affinity receptors rather than the low affinity Fc receptors engaged by ICs. These differences suggest that the isotype control antibody will lack the ability to induce correlate Fc functions to the experimental antibody. As such, IgG isotypes are probably not appropriate controls for antibody-based studies that employ functional Fc domains.

## Closing Thoughts and Future Directions

The development of recombinant Fc multimers as treatments for autoimmune and inflammatory conditions provides an opportunity for the scientific community to reconsider the role of ICs in inflammation. Specifically, the data that ICs induce tolerance should prompt consideration of how/if fully competent monoclonal antibodies used for treatment of cancer and autoimmunity, may paradoxically mediate tolerogenic effects when decorating the target cell surface. Similarly, taken in concert, these data raise the possibility that the initial characterizations of ICs as pathogenic were perhaps oversimplified and that, in fact, the function of naturally occurring ICs are largely dependent on their antigenic core. Building on this theme, is it also possible, that some ICs are necessary sequelae of inflammatory events and that, under specific conditions, they play an important role in restoring immune homeostasis. Existing recombinant ICs, in combination with development of more specific agents—through altered N-glycosylation, sequence modifications, and valency specific selection—will help the scientific community address these basic questions that are fundamental to our understanding of inflammation.

## Author Contributions

EF, JW, and SS wrote sections of the manuscript. EF revised and edited the final document and generated figures. SS contributed critical input to revisions.

### Conflict of Interest

SS is a cofounder, paid consultant, stockholder, and has received research support from Gliknik Inc., a biotechnology company. His laboratory has received research support from Pfizer Inc. through a sponsored research agreement. He also receives royalties from intellectual property related to B7-H1(PD-L1): PD-1 licensed by the Mayo Clinic College of Medicine to third parties. He sits on the scientific advisory board of Virion Inc. The remaining authors declare that the research was conducted in the absence of any commercial or financial relationships that could be construed as a potential conflict of interest.
